# Supplementary material for: WordCommentsAnalyzer: A windows software tool for qualitative research
Source: F1000Res. 2018 Sep 4;7:536. Originally published 2018 May 3. [Version 2] doi: 10.12688/f1000research.14819.2 (PMC6137411; doi:10.12688/f1000research.14819.2)
Supplement: Supplementary file 3 [file f1000research-7-17614-s0002.tgz › b2a79496-4ada-47c4-9682-a99ce867836d.zip!/Eight Iranian Health Journals/Journal of Research in Rehabilitation Sciences _32.docx]

© Journal of Research in Rehabilitation Sciences . This work is licensed under CC BY-NC 4.0 (https://creativecommons.org/licenses/by-nc/4.0/).

Volume & Issue: دوره 9، شماره 1: 1392

Title: بررسی ارتباط میزان تمایل لگن با عملکرد و طول عضلات در زنان سالم

http://jrrs.mui.ac.ir/index.php/jrrs/article/view/827

مقدمه: تيلت لگن (Pelvic inclination) با تمايل لگن نسبت به صفحه افقي تعريف مي‌شود. از جمله عوامل موثر بر تیلت لگن می‌توان به میزان انعطاف‌پذیری و قدرت عضلات ناحیه کمری- لگنی اشاره کرد. مطالعاتی که ارتباط بین تیلت لگن، طول و عملکرد عضلات را بررسی کرده‌اند، نتایج متناقضی داشته‌اند. هدف مطالعه حاضر، بررسی رابطه میان این متغیرها در یک نمونه بزرگ و با ابزارهای دقیق‌تر بود. مواد و روش‌ها: در این مطالعه 180 زن سالم (65-20 سال) شرکت کردند. تیلت لگن به وسیله تیلت‌سنج و عملکرد عضلانی با استفاده از دو روش ثبت قدرت ایزومتریک با دینامومتر و ثبت تحمل با کرونومتر اندازه‌گیری گردید. میزان انعطاف پذیری هر عضله با تست‌های کوتاهی قراردادی اختصاصی تست شد. یافته‌ها: بین تیلت لگن و طول عضله ایلیوپسواس (33/0= r)، قدرت عضلات اکسترنال (39/0 = r) و اینترنال روتاتور ران (32/0 = r) ارتباط ضعیف معنی‌داری وجود داشت. همچنین ارتباط معنی‌دار ضعیفی بین تیلت لگن و تحمل عضلات فلکسور (20/0 = r) و فلکسور طرفی تنه (22/0 = r) مشاهده شد. در بررسی این ارتباطات به تفکیک گروه‌های سنی (صرف نظر از تفاوت‌های مختصر در گروه‌های سنی مختلف) نتایج مشابه نتایج در کل نمونه‌ها بود. نتیجه‌گیری: با توجه به نوع چسبندگی عضلات ناحیه لومبوپلویک به لگن، به نظر می‌رسد که تغییرات طول و عملکرد عضلات این ناحیه بتواند بر نحوه قرارگیری آن مؤثر باشد، اما در مطالعه حاضر وجود این تأثیر ثابت نشد. بسیاری از این عضلات دو یا چند مفصلی می‌باشند، بنابراین شاید اثر تغییرات قدرت یا تحمل و طول خود را در مناطقی دیگر (غیر از لگن) نشان دهند. همچنین چون افراد این مطالعه از نظر انعطاف‌پذیری و عملکرد عضلانی در محدوده غیر نرمال نبودند شاید عاملی برای این ارتباط ضعیف یا عدم ارتباط باشد.

Title: بررسی مهارت‌های شناختی، شنیداری، زبانی و گفتاری در کودکان دارای شکاف کام و لب

http://jrrs.mui.ac.ir/index.php/jrrs/article/view/901

مقدمه: شکاف کام و لب جنبه‌های رشدی کودک مانند مهارت‌های شناختی، شنیداری، زبانی و گفتاری را متأثر می‌سازد. تأخیر در تشخیص و ارزیابی همه جانبه این کودکان تأثیر غیر قابل جبرانی بر آن‌ها خواهد گذاشت. به همین دلیل در این مطالعه، مهارت‌های رشدی در کودکان دارای شکاف کام و لب مورد بررسی قرار گرفت. مواد و روش‌ها: طی مطالعه توصیفی- تحلیلی حاضر از نوع شاهد موردی، تعداد 72 کودک 6-3 ساله در قالب دو گروه، 40 کودک هنجار و 32 کودک دارای شکاف کام و لب در چهار رده سنی 72-61، 60-49، 48-43 و 42-37 ماهه با استفاده از آزمون سنجش رشد مهارت‌های شناختی، شنیداری، گفتاری، زبان دریافتی و بیانی نیوشا مورد ارزیابی قرار گرفتند. یافته‌ها: میانگین امتیازات مهارت‌های زبان- بیانی و مهارت گفتاری در دو گروه در محدوده‌های سنی مختلف با هم تفاوت معنی‌داری داشتند (05/0 > P). همچنین سابقه ابتلا به عفونت گوش میانی در کودکان دارای شکاف کام و لب بیشتر از گروه هنجار بود. نتیجه‌گیری: مهارت‌های رشدی در کودکان دارای شکاف کام و لب تحت تأثیر نقص ساختاری موجود قرار می‌گیرد. ارزیابی جامع و زودهنگام این کودکان می‌تواند به طراحی دقیق برنامه مداخله‌ای کمک کند.

Title: بررسي ارتباط بین قدرت عضلات اندام تحتانی و تعادل پویا در بیماران زن مبتلا به مولتیپل اسکلروزیس

http://jrrs.mui.ac.ir/index.php/jrrs/article/view/594

مقدمه: مشکلات تعادل و کنترل وضعیت بدن در جمعیت افراد مبتلا به مولتیپل اسکلروزیس (Multiple sclerosis یا MS) مسأله شایعی است؛ به طوری که میزان خطر شکستگی ناشی از به زمین افتادن در این افراد 2 تا 3 برابر افراد سالم می‌باشد. هدف از این مطالعه، بررسي رابطه بین قدرت عضلانی اندام تحتانی و تعادل پویا در بیماران مبتلا به MS بود. مواد و روش‌ها: تعادل پویای 33 نفر از افراد مبتلا به MS توسط آزمون تعادلی Berg (Berg balance scale یا BBS)، آزمون بلند شدن و رفتن زمان‌دار (Timed up and go test یا TUG) و تست رسیدن عملکردی (Functional reach test یا FRT) سنجیده شد. همچنین قدرت عضلات اندام تحتانی دو طرف با استفاده از دینامومتر و تست 30 ثانیه نشست و برخاست اندازه‌گیری شد. یافته‌ها: بین قدرت عضلات اندام تحتانی با FRT رابطه وجود داشت (01/0 > P). همچنین تست TUG با قدرت عضلات اکستنسور و فلکسور زانو در سطح 05/0 > P و با تست 30 ثانیه نشست و برخاست در سطح 01/0 > P رابطه داشت. رابطه بین آزمون تعادلی Berg و قدرت اکستنسورها (راست 47/0 = r و چپ 46/0 = r، 01/0 > P) و فلکسورهای زانو (راست 45/0 = r، 01/0 = P و چپ 37/0 = r، 05/0 > P) معنی‌دار بود؛ در حالی که رابطه بین این آزمون تعادلی با تست 30 ثانیه نشست و برخاست در سطح 01/0 = P معنی‌دار بود. نتیجه‌گیری: به طور کلی نتایج حاصل از این مطالعه نشان داد که کاهش قدرت عضلانی اندام تحتانی می‌تواند بر تعادل دینامیک بیماران مبتلا به MS اثرگذار باشد. این نتایج برای ارزیابی کیفیت برنامه توان‌بخشی ویژه بیماران MS مفید هستند. پیشنهاد می‌شود به منظور بهبود تعادل و کاهش خطر افتادن در این بیماران، افزایش قدرت عضلانی اندام تحتانی مورد توجه ویژه قرار گیرد.

Title: تأثیر تمرینات پیلاتس و کینزیوتیپ بر درد و ناتوانی عملکردی مردان مبتلا به کمردرد مزمن غیر اختصاصی

http://jrrs.mui.ac.ir/index.php/jrrs/article/view/702

مقدمه: کمردرد مزمن یک مشکل رو به رشد در بسیاری از جوامع است، اما در مورد مناسب‌ترین مداخله درمانی توافقی وجود ندارد. به تازگی تمرینات پیلاتس که روشی برای سلامتی ذهن و جسم می‌باشد به عنوان یک روش بازتوانی پذیرفته شده است. همچنین یکی دیگر از روش‌های درمانی جدید کینزیوتیپینگ (Kinesio taping) است که ادعا می‌شود قادر به کاهش درد، تورم و اسپاسم عضلانی است و در ارتوپدی، طب ورزشی و توان‌بخشی عصبی- عضلانی متداول شده است. بنابراین هدف از پژوهش حاضر، بررسی تأثیر تمرینات پیلاتس و کینزیوتیپ بر شدت درد و ناتوانی عملکردی مردان مبتلا به کمردرد مزمن غیر اختصاصی بود. مواد و روش‌ها: 24 مرد مبتلا به کمردرد مزمن غیر اختصاصی با میانگین سنی 82/6 ± 54 سال به عنوان نمونه به صورت هدفمند انتخاب و به دو گروه 12 نفره تقسیم شدند. گروه اول تمرینات پیلاتس را انجام دادند و گروه دوم با استفاده از کینزیوتیپ، نواربندی (Taping) شدند. طول مدت درمان برای هر دو گروه 6 هفته بود. از مقیاس VAS (Visual analog scale) برای ارزیابی تغییرات شدت درد و از مقیاس ناتوانی عملکردی Quebec (Quebec functional disability scale) جهت ارزیابی ناتوانی عملکردی استفاده شد. برای تحلیل داده‌ها از تحليل واريانس اندازه‌هاي تكراري با سطح معنی‌داری 05/0 £ P استفاده گردید. یافته‌ها: بهبود معنی‌داری در شدت درد و ناتوانی عملکردی در هر دو گروه پس از درمان دیده شد (001/0 > P). از طرفی، مقایسه داده‌ها پس از مداخله اختلاف معنی‌داری در شدت درد بین دو گروه را نشان داد (001/0 > P)، اما اختلاف معنی‌داری از نظر ناتوانی عملکردی بین دو گروه دیده نشد (188/0 = P). نتیجه‌گیری: تمرینات پیلاتس نسبت به کینزیوتیپ، اثرات بهتری را در کاهش درد و افزایش سطح عملکرد مبتلایان به کمردرد مزمن غیر اختصاصی ایجاد می‌کند.

Title: تأثیر هشت هفته تمرینات یوگای خنده بر عملکرد حرکتی، تعادل و انعطاف‌پذیری بیماران مبتلا به پارکینسون

http://jrrs.mui.ac.ir/index.php/jrrs/article/view/856

مقدمه: بیماری پارکینسون یکی از شایع‌ترین بیماری‌های اختلال در سیستم اعصاب مرکزی است که باعث کندی حرکات، لرزش، بی‌ثباتی وضعیت قامت، کاهش تعادل و اختلالات سیستم اعصاب خودکار می‌شود. یوگای خنده یکی از درمان‌های مکمل و ترکیبی از حرکات کششی و تنفسی یوگا به همراه خنده می‌باشد. هدف این پژوهش، بررسی تأثیر تمرینات یوگای خنده بر عملکرد حرکتی، تعادل و انعطاف‌پذیری بیماران زن مبتلا به پارکینسون بود. مواد و روش‌ها: تعداد 24 زن مبتلا به بیماری پارکینسون (مرحله 3-1 بر اساس مقیاس Hoehn و Yahr) به صورت هدفمند و داوطلبانه انتخاب و سپس به صورت تصادفی در دو گروه تجربی (انحراف معیار ± میانگین) (سن 8/6 ± 6/52 سال، وزن 8/7 ± 1/61 کیلوگرم و قد 3/4 ± 8/157 سانتی‌متر) و شاهد (سن 7/6 ± 5/55 سال، وزن 8/6 ± 67 کیلوگرم و قد 5/5 ± 4/158 سانتی‌متر) قرار گرفتند. گروه تجربی علاوه بر درمان‌های دارویی به مدت 8 هفته، هر هفته 3 جلسه، هر جلسه 45 دقیقه به انجام فعالیت یوگای خنده پرداختند؛ در حالی که بیماران گروه شاهد در این مدت جز درمان‌های دارویی، هیچ فعالیت بدنی مؤثری را انجام ندادند. در ابتدا و انتهای تحقیق عملکرد حرکتی (قسمت 3 پرسش‌نامه UPDRS)، تعادل (Berg) و انعطاف‌پذیری [تست بشین و برس (سانتی‌متر)] بیماران ارزیابی شدند و داده‌ها با استفاده از آزمون Independent t مورد تجزیه و تحلیل قرار گرفت. یافته‌ها: یافته‌های تحقیق بهبود معنی‌داری (05/0 > P) را در عملکرد حرکتی بیماران گروه تجربی بعد از هشت هفته مداخله نشان داد (9/12 در مقابل 6/12)؛ به طوری که عملکرد حرکتی بیماران گروه تجربی حدود 2 درصد بهبود یافت؛ در حالی که در بیماران گروه شاهد کاهش معنی‌داری (05/0 > P) مشاهده شد (3/14 در مقابل 7/14). اگرچه تعادل در بیماران گروه تجربی تا حدي بهبود يافت، اما اين تغيير از لحاظ آماري معني‌دار نبود (05/0 < P). در متغیر انعطاف‌پذیری افزایش معنی‌داری (05/0 > P) در بیماران گروه تجربی مشاهده شد (1/11 در مقابل 3/13) و در گروه شاهد کاهش معنی‌داری (05/0 > P) یافت شد (13 در مقابل 5/12). در متغیرهای ذکر شده بین دو گروه تجربی و شاهد تفاوت معنی‌داری مشاهده شد (05/0 > P). نتیجه‌گیری: تمرینات یوگای خنده تأثیر مثبتی بر عملکرد حرکتی و انعطاف‌پذیری بیماران مبتلا به پارکینسون دارد، بنابراین یوگای خنده به عنوان یک مدالیته مفید برای این دسته از بیماران توصیه می‌شود.

Title: بررسی تأثیر آموزش مراقبت‌های ویژه بر استرس و کیفیت زندگی مادران کودکان فلج مغزی 12-4 ساله شهر تهران

http://jrrs.mui.ac.ir/index.php/jrrs/article/view/862

مقدمه: وجود کودک مبتلا به فلج مغزی، مادران را در معرض استرس‌های مراقبتی قرار می‌دهد و کیفیت زندگی آنان را در تمامی ابعاد متأثر می‌سازد. بنابراین پژوهش حاضر با هدف، تعیین میزان تأثیر برنامه آموزش مراقبت‌های ویژه بر استرس و کیفیت زندگی مادران کودکان فلج مغزی انجام گردید. مواد و روش‌ها: این مطالعه از نوع کارآزمایی بالینی بود که با انتخاب تصادفی دو گروه آزمون (29 نفر) و شاهد (25 نفر) انجام شد. ابزارهای جمع‌آوری اطلاعات شامل پرسش‌نامه منابع و استرس، پرسش‌نامه کیفیت زندگی 36-SF (Short form-36) و اطلاعات دموگرافیک بود. داده‌ها قبل و سه ماه بعد از مداخله جمع‌آوری و توسط نرم‌افزار SPSS نسخه 16 مورد تجزیه و تحلیل قرار گرفت. یافته‌ها: ميانگين سن مادران در گروه شاهد 9/4 ± 8/32 سال و در گروه آزمایش 4/4 ± 3/35 سال بود و ميانگين سن كودكان در گروه شاهد 1/2 ± 2/6 سال و در گروه آزمايش 8/2 ± 0/7 سال بود. آزمون آماری Independent t بین ابعاد کیفیت زندگی در دو گروه آزمون و شاهد بعد از مداخله، اختلاف معنی‌داری را نشان داد (05/0 > P)، اما تنها در دو خرده مقیاس عملکرد جسمی و سلامت عاطفی اختلاف معنی‌دار یافت نشد (05/0 < P). همچنین در تمامی ابعاد استرس اختلاف معنی‌داری بین دو گروه رؤیت گردید (05/0 > P). نتیجه‌گیری: اجرای برنامه آموزش مراقبت‌های ویژه بر کاهش استرس و ارتقای کیفیت زندگی مادران کودکان فلج مغزی موثر می‌باشد.

Title: شناخت اجتماعي و کنترل حرکتي: شواهدي از اثر مشاهده وضعيت تعادلي بر نوسان وضعيتي

http://jrrs.mui.ac.ir/index.php/jrrs/article/view/591

مقدمه: نورون‌هاي آينه‌اي دسته‌اي از نورون‌ها هستند كه هنگام انجام يك حركت و نيز در هنگام مشاهده انجام حركت توسط ديگري، فعال مي‌شوند. هدف پژوهش حاضر، بررسي اثر مشاهده وضعيت تعادلي بر نوسان وضعيتي بود. مواد و روش‌ها: در اين مطالعه مقطعي، عملکرد وضعيتي 20 نفر از دانشجويان با استفاده از دستگاه تعادل بايودکس در سه شاخص نوسانات در راستاي داخلي- خارجي، نوسانات در راستاي قدامي- خلفي و نوسانات کلي در هنگام مشاهده و عدم مشاهده وضعيت تعادلي بررسي گرديد. جهت مقايسه ميانگين نوسانات در دو حالت، از آزمون Paired-t استفاده گرديد. یافته‌ها: در دو پارامتر نوسان در راستاي داخلي- خارجي و کلي، مقايسه دو حالت مقايسه‌ ميانگين‌ها نشان دهنده‌ تفاوت معنی‌دار بود (018/0 = P، 049/0 = P) اما در پارامتر نوسان در راستاي قدامي- خلفي، تفاوت ميانگين‌ها به حد معنی‌دار نرسيده بود (049/0 = P). نتیجه‎گیری: بر اساس نتايج به دست آمده، مشاهده فرد بي‌ثبات، باعث افزايش نوسانات وضعيتي مي‌شود. اين يافته‌ها مي‌تواند با فعاليت نورون‌هاي آينه‌اي درگير در کنترل حرکتي در ارتباط باشد. مي‌توان بر اساس اين يافته پيشنهاد نمود که انجام تکليف تعادلي به همراه بيمار مي‌تواند به عنوان يک اضافه بار براي تکليف حرکتي او باشد.

Title: مقایسه اثر دو تکنیک ماساژ درمانی و استرین– کانتراسترین بر درد و عملکرد مبتلایان به گردن‌‌درد

http://jrrs.mui.ac.ir/index.php/jrrs/article/view/624

مقدمه: گردن‌درد یکی از ضایعات شایع اسکلتی- عضلانی می‌باشد و عدم درمان مناسب این عارضه می‌تواند سبب ایجاد ناتوانی برای افراد شود. درمان‌های دستی گروهی از درمان‌های کم‌هزینه، با حداقل عوارض جانبی هستند که در درمان گردن‌درد مورد استفاده قرار می‌گیرند، اما مطالعات کمی در زمینه تأثیر این درمان‌ها بر گردن‌درد و یا مقایسه آن‌ها جهت یافتن درمان مؤثرتر وجود دارد. هدف از این تحقیق، مقایسه تأثیر دو تکنیک دستی شامل ماساژ درمانی و استرین- کانتراسترین بر کاهش درد و بهبود عملکرد در افراد مبتلا به گردن درد مکانیکال بود. مواد و روش‌ها: تحقیق حاضر بر روی 30 زن مبتلا به گردن‌درد در محدوده سنی 35-18 سال انجام شد. بیماران به صورت تصادفی به دو گروه تقسیم شدند. گروه اول، ماساژ درمانی و گروه دوم، استرین- کانتراسترین دریافت نمودند. هر گروه، سه جلسه درمان را یک روز در میان دریافت کردند. درد بر اساس مقیاس عددی شاخص درد (Pain numerical rating scale یا PNRS) و سطح ناتوانی عملکردی بیمار بر اساس پرسش‌نامه شاخص ناتوانی گردن (Neck disability index یا NDI)، قبل و بلافاصله بعد از درمان ارزیابی شد. روش‌های آماری مورد استفاده شامل آزمون Paired t و Independent t بود. یافته‌ها: اختلاف معنی‌داری در شدت درد و سطح ناتوانی عملکردی بعد از ماساژ درمانی بر اساس مقیاس عددی سنجش درد (001/0 = PNRS) و پرسشنامه سطح ناتوانی عملکردی گردن (0 = PNDI) و بعد از درمان استرین-کانتراسترین (03/0 = PNDI و 0 = PNRS) مشاهده شد. در مقایسه دو گروه پس از درمان، در متغیر شدت درد اختلاف معنی‌داری مشاهده گردید (001/0 > P)، اما در سطح ناتوانی عملکردی اختلاف معنی‌داری بین دو گروه مشاهده نگردید (057/0 = P). نتیجه‌گیری: ماساژ درمانی و استرین- کانتراسترین هر دو در کاهش درد و بهبود عملکرد بیماران مبتلا به گردن‌درد مؤثر هستند. تکنیک استرین- کانتراسترین در مقایسه با ماساژ درمانی تأثیر بیشتری در کاهش درد داشت.

Title: بررسی رابطه بین شاخص‌های کتف و پکتورالیس مینور پس از یک دوره تمرین درمانی در افراد شانه به جلو

http://jrrs.mui.ac.ir/index.php/jrrs/article/view/566

مقدمه: تغییرات در کمربند شانه و کتف به هم مرتبط هستند و بیان شده است که انحراف وضعیتی کمربند شانه با عدم تعادل عضلانی اطراف شانه هم‌زمان است. هدف از تحقیق حاضر، بررسی رابطه بین شاخص‌های کتف و پکتورالیس مینور پس از یک دوره تمرینات منتخب کششی- قدرتی در افراد شانه به جلو بود. مواد و روش‌ها: بر این اساس افراد با وضعیت شانه به جلو با استفاده از صفحه شطرنجی از بین دانش‌آموزان دختر 12-9 ساله غربالگری شده و از بین آن‌ها 20 نفر به صورت تصادفی گزینش شدند. پس از تکمیل فرم‌های پرسش‌نامه و رضایت‌نامه، وضعیت شانه به جلو با دستگاه چهارگوش دوگانه (فاصله زایده آخرومی شانه از دیوار)، فاصله بین نقطه میانی حفره جناغی با زایده غرابی و فاصله افقی لبه خلفی جانبی آخروم با ستون فقرات به منظور اندازه‌گیری شاخص کتف، فاصله بین نقطه میانی حفره جناغی با زایده غرابی و نقطه میانی دنده‌های سوم تا پنجم (طول عضله پکتورالیس مینور) و قد آزمودنی‌ها به منظور اندازه‌گیری شاخص پکتورالیس مینور با متر نواری اندازه‌گیری گردید. آزمودنی‌ها با توجه به فاصله شانه از دیوار، به گروه‌های همگن شاهد و تجربی تفکیک شدند و سپس گروه تجربی تمرینات منتخب 6 هفته‌‌ای را اجرا کردند. یافته‌ها: تجزیه و تحلیل اطلاعات نشان داد که تمرین در گروه تجربی باعث کاهش معنی‌دار 12 درصدی ناهنجاری شانه به جلو (008/0 = P)، افزایش معنی‌دار فاصله SN (Sternal notch) به CP (Coracoied prossess) (024/0 = P)، بهبود معنی‌دار شاخص پکتورالیس مینور (019/0 = P) و شاخص کتف (023/0 = P) شد. تأثیر معنی‌داری در فاصله PLA (Posterolateral acromion) به TS (Thoracic spine) مشاهده نشد و رابطه معنی‌داری بین شاخص‌های کتف و پکتورالیس مینور (080/0 = P) وجود داشت. نتیجه‌گیری: اجرای یک دوره تمرین درمانی موجب بهبود وضعیت شانه، شاخص کتف و شاخص پکتورالیس مینور افراد شانه به جلو می‌شود و همچنین شاخص کتف و شاخص پکتورالیس مینور دارای همبستگی مثبت بالایی می‌باشند.

Title: مقایسه نسبت قدرت و دامنه حرکتی چرخش داخلی به خارجی مفصل شانه ورزشکاران زورخانه با افراد غیر ورزشکار

http://jrrs.mui.ac.ir/index.php/jrrs/article/view/641

مقدمه: عضلات روتیتورکاف که مسؤول چرخش داخلی و خارجی بازو هستند، نقشی حیاتی در ایجاد حرکت‌پذیری و پایداری مفصل شانه دارند. این وظیفه در ورزشکارانی که نیاز به اجرای حرکات مکرر در بالای سر دارند، دارای اهمیت بیشتری است. از آن‌جایی که تطابق اسکلتی- عضلانی به واسطه بعضی نیازهای فیزیکی و الگوهای حرکتی برای ورزشکاران بسیار مهم است، هدف از تحقیق حاضر، مقایسه نسبت قدرت و دامنه‌ حرکتی چرخش داخلی و خارجی مفصل شانه ورزشکاران زورخانه با افراد غیر ورزشکار بود. مواد و روش‌ها: آزمودنی‌های این تحقیق شامل 29 ورزشکار زورخانه با میانگین سنی 04/2 ± 51/23 سال و میانگین سابقه ورزشی 47/4 ± 75/8 سال و 30 نفر غیر ورزشکار با میانگین سنی 56/1 ± 8/24 سال بودند. جمع‌آوری اطلاعات از طریق پرسش‌نامه و معاینه صورت‌ گرفت. قدرت ایزومتریک توسط دستگاه قدرت‌سنج دستی و دامنه حرکتی توسط انعطاف‌سنج لیتون در حرکات چرخش داخلی و خارجی هر دو اندام ‌فوقاني اندازه‌‌گیری شدند. از آزمون Independent t برای تجزیه تحلیل داده‌ها استفاده‌ گردید. یافته‌ها: نسبت دامنه حرکتی چرخش داخلی به خارجی مفصل شانه در ورزشکاران زورخانه کمتر از افراد غیر ورزشکار بود (050/0 ≥ P). به علاوه، نتایج مؤید آن است که نسبت قدرت چرخش داخلی به خارجی ورزشکاران زورخانه در این مفصل بیشتر از گروه غیر ورزشکار می‌باشد (001/0 ≥ P). نتیجه‌گیری: انجام الگوهای ‌حرکتی ‌تکراری در زورخانه، ممکن است در مدت زمان طولانی باعث به هم خوردن نسبت طبیعی تعادل قدرت و دامنه حرکتی در مفصل شانه شود که به عنوان عامل خطرزا در آسیب‌های شانه، مستلزم توجه به رویکردهای پیشگیرانه با ایجاد راهکارهای اصلاحی می‌باشد.

Title: مقایسه تأثیر تکلیف بینایی بر کنترل وضعیتی در بیماران همی‌پارزی مزمن راست و چپ (مطالعه مقدماتی)

http://jrrs.mui.ac.ir/index.php/jrrs/article/view/893

مقدمه: نقش و اهمیت تکالیف دوگانه و نیم‌کره راست برای حفظ کنترل وضعیتی، در مطالعات بسیاری ذکر گردیده است، اما تأثیر سطوح مختلف تکلیف بینایی روی کنترل وضعیتی در بیماران همی‌پارزی مزمن راست و چپ مشخص نشده است. بنابراین این مطالعه با هدف، بررسی مقایسه‌ای تأثیر تکلیف بینایی بر کنترل وضعیتی بیماران همی‌پارزی مزمن راست و چپ انجام شد. مواد و روش‌ها: 6 بیمار همی‌پارزی راست و 14 بیمار همی‌پارزی چپ به روش نمونه‌گیری غیر احتمالی ساده در مطالعه حاضر شرکت کردند. کنترل وضعیتی بر روی صفحه نیرو در حالات مختلف چشم باز، چشم بسته، جستجوی هدف‌دار در فاصله دور و نزدیک و جستجوی بدون هدف در فاصله دور و نزدیک مورد بررسی قرار گرفت. پارامترهای نوسان مرکز فشار در این مطالعه شامل سطح کل نوسان، میانگین سرعت، طول مسیر و سطح فاز کل بود. یافته‌ها: اثر اصلی تکلیف وضعیتی در تمامی پارامترها (میانگین سرعت، طول مسیر و سطح فاز کل) به جز سطح کل نوسان و همچنین اثر اصلی سمت مبتلا فقط در پارامتر سطح کل نوسان مرکز فشار معنی‌دار بود (05/0 £ P). اثر متقابل سمت مبتلا با تکلیف وضعیتی در هیچ یک از پارامترهای مرکز فشار به جز صفحه فاز کل معنی‌دار نبود (05/0 ³ P). نتیجه‌گیری: در هر دو گروه بیماران همی‌پارزی مزمن راست و چپ، فاصله تکلیف بینایی بر کنترل وضعیتی تأثیر داشت، ولی نوع تکلیف بینایی تأثیر معنی‌داری روی کنترل وضعیتی نداشت.

Title: تأثیر روش تمرین درمانی و ارگونومیک مبتنی بر فضای مجازی بر روی کاربران رایانه مبتلا به کمردرد

http://jrrs.mui.ac.ir/index.php/jrrs/article/view/743

مقدمه: کمردرد یکی از شایع‌ترین آسیب‌های مرتبط با محیط کار می‌باشد که در سال‌های اخیر با وجود به کارگیری روش‌های درمانی حضوری، روش‌های مبتنی بر تکنولوژی‌های جدید برای آن معرفی نشده است. بنابراین هدف این پژوهش، مقایسه تأثیر دو روش تمرین درمانی و ارگونومیک مبتنی بر فضای مجازی بر روی کاربران رایانه مبتلا به کمردرد بود. مواد و روش‌ها: در پژوهش نیمه تجربی حاضر تعداد 150 بیمار مبتلا به کمردرد، به صورت تصادفی در دو گروه شاهد (ارگونومیک) و آزمایش (ترکیبی شامل تمرین درمانی و ارگونومیک) قرار گرفتند. هر دو گروه، درمان خود را از طریق فضای مجازی دریافت نمودند. گروه شاهد، از طریق مداخله‌های درمانی ارگونومیک و گروه آزمایش علاوه بر مداخله‌های درمانی ارگونومیک، به تمرین درمانی نیز پرداخت. میزان درد و ناتوانی از طریق پرسش‌نامه اصلاح شده Nordic قبل و بعد از سه ماه مداخله مورد ارزیابی قرار گرفت. جهت تحلیل یافته‌ها از آزمون 2c استفاده گردید. یافته‌ها:درد کمر در افراد مبتلا به ناهنجاری کمر در گروه آزمایش نسبت به گروه شاهد در ارزیابی 12 ماهه و 7 روز بعد از آن به طور معنی‌داری کاهش یافت (05/0 > P). نتیجه‌گیری: استفاده از روش تمرین درمانی و ارگونومیک مبتنی بر فضای مجازی موجب بهبود کمردرد مزمن در کاربران رایانه می‌شود، بنابراین انجام این نوع از تمرینات توصیه می‌گردد.

Title: بررسی تأثیر تمرینات قدرتی، تعادلی و ترکیبی (قدرتی و تعادلی) بر تعادل پویای نوجوانان پسر ورزشکار

http://jrrs.mui.ac.ir/index.php/jrrs/article/view/898

مقدمه: با افزایش روزافزون زمینه‌های ورزشی تعداد نوجوانانی که در فعالیت‌های ورزشی دچار آسیب می‌شوند در حال افزایش است و بیشترین میزان این آسیب‌ها در این گروه سنی اتفاق می‌افتد. هدف از پژوهش حاضر، بررسی تأثیر شش هفته تمرین قدرتی، تعادلی و ترکیبی بر تعادل پویای نوجوانان پسر ورزشکار بود. 60 نوجوان پسر ورزشکار 17-14 سال با میانگین و انحراف استاندارد وزنی 62/3 ± 79/62 کیلوگرم و قد 46/4 ± 1/171 سانتی‌متر، بدون سابقه آسیب در اندام تحتانی به صورت تصادفی به چهار گروه 15 نفری شامل گروه‌های تمرینی قدرتی، تعادلی و ترکیبی (قدرتی و تعادلی) و گروه شاهد تقسیم شدند. مواد و روش‌ها: قبل و بعد از اجرای شش هفته برنامه تمرینی، تعادل پویای آزمودنی‌ها با تست تعادل ستاره اندازه‌گیری شد. برای تجزیه و تحلیل داده‌ها از روش‌های آمار توصیفی، آزمون اندازه‌گیری‌های تکراری، آزمون Paired t، ANOVA و آزمون تعقیبی Tukey در سطح معنی‌داری 05/0 ≤ α استفاده شد. یافته‌ها: تمرین قدرتی، تعادلی و ترکیبی، افزایش معنی‌دار فاصله دستیابی آزمودنی‌ها در هر هشت جهت را نشان دادند و همچنین تمرین ترکیبی از تمرین قدرتی و تعادلی و تمرین تعادلی نیز از تمرین قدرتی بهبود بیشتری در تعادل پویای آزمودنی‌ها ایجاد می‌کند. نتیجه‌گیری: با توجه به نیاز ویژه نوجوانان به تعادل در مهارت‌های ورزشی و همچنین به عنوان عامل مهم در پیشگیری از آسیب‌های ورزشی، بهتر است در طراحی برنامه‌های تمرینی از مزایای هر سه برنامه تمرین به ویژه تمرینات ترکیبی استفاده کنند.

Title: مروری بر ارتباط بین درد، ترس از درد و ثبات پوسچر در بیماران مبتلا به کمردرد مزمن غیر اختصاصی

http://jrrs.mui.ac.ir/index.php/jrrs/article/view/659

مقدمه: کمردرد از شایع‌ترین بیماری‌ها در کشورهای پیشرفته و در حال پیشرفت است که 70 تا 80 درصد بالغین در زندگی خود به آن مبتلا می‌شوند. مطالعات نشان داده است که عوامل روحی- روانی به ویژه ترس از درد در تعیین بیمارانی که درد آن‌ها مزمن می‌شود و یا درمان آن‌ها موفقیت‌آمیز نیست، نقش مهمی دارند. هدف این مطالعه توصیفی، مروری بر مطالعاتی است که ارتباط بین درد و ترس از درد را با ثبات پوسچرال در بیماران مبتلا به کمردرد مزمن غیر اختصاصی (Chronic nonspecific low back pain یا CLBP) بررسی نموده‌اند. مواد و روش‌ها: در مطالعه مروری- توصیفی حاضر، با استفاده از کلید واژه‌های «کمردرد، کمردرد مزمن، ترس از درد، پوسچر، ثبات، کنترل حرکتی، مرکز ثقل (Center of pressure) و صفحه نیرو» پایگاه‌های اطلاعاتی PubMed و  Medline را جستجو نمودیم. شاخص‌های ورود شامل: مقالات مرتبط با ترس از درد و ثبات پوسچرال در بیمارن بالغ مبتلا به کمردرد مزمن غیر اختصاصی، به زبان انگلیسی تا سال 2011 در غالب مطالعات مقطعی و کوهورت بود. یافته‌ها: عوامل روحی- روانی از جمله ترس از درد، در مزمن شدن کمردرد تأثیر دارند. از نظر ارتباط درد با ثبات پوسچرال نتایج متفاوت بود. تنها یک مطالعه ارتباط ترس از درد و ثبات پوسچرال را بررسی کرده بود که در آن ارتباط معنی‌داری بین این دو پارامتر یافت نشده بود. نتیجه‌گیری: به نظر می‌رسد تفاوت نتایج مطالعات مختلف ناشی از تفاوت در روش جمع‌آوری داده‌ها باشد. مطالعات بیشتر و دقیق‌تری برای بررسی ارتباط ترس از درد و ثبات پوسچرال نیاز است.

Title: بررسی تاثیرسیلیکون لاینر ها بر کیفیت راه رفتن افراد مبتلا به قطع عضو زیر زانو ؛ مروری بر مقالات

http://jrrs.mui.ac.ir/index.php/jrrs/article/view/929

مقدمه: قطع عضوهای اندام تحتانی به طور معمول به علت ضربه، بیماری‌های عروقی، دیابت و یا اختلالات مادرزادی ایجاد می‌شود. افراد دچار قطع عضو (Amputee)، توانایی ایستادن و راه رفتن را با توجه به سطح قطع عضو خود از دست می‌دهند.‌ انواع مختلفی از پروتزها جهت افزایش عملکرد افراد قطع عضو مورد استفاده قرار می‌گیرد. کارآمدی پروتزهای اندام تحتانی توسط یک روش تعلیق مناسب تعیین می‌گردد، در حقیقت تعلیق و Fitting مناسب نقش مهمی در راحتی و عملکرد پروتز ایفا می‌نماید. به طور تقریبی سه دهه از پیدایش پروتزهای سیلیکون لاینر می‌گذرد. تأثیر این نوع سیستم پروتزی بر کارایی پروتز به طور واضح مشخص نشده است. علاوه بر آن، تأثیر سیلیکون لاینر بر عواملی مانند سرعت راه رفتن، تعادل ایستگاهی و اطمینان راه رفتن مشخص نیست. بنابراین هدف از این مقاله مروری، بررسی تأثیر سیلیکون لاینر بر پارامترهای ذکر شده در بالا با توجه به تحقیقات موجود بود. مواد و روش‌ها: یک جستجوی الکترونیک در پایگاه‌های اطلاعاتی PubMed، ISI web of science و Embase از سال 1960 تا 2012 و با استفاده از کلمات کلیدی Icelandic roll on silicon، Trans-tibialamputation، Socket (ICEROSS)، Trans-tibial prosthesis و Silicone liner انجام گردید و کیفیت مقالات با استفاده از ابزار Black and down مورد ارزیابی قرار گرفت. یافته‌ها: بر اساس کلمات کلیدی استفاده شده، 100 مقاله به دست آمد که 10 مقاله با توجه به معیارهای انتخاب برای تحلیل نهایی پذیرفته شدند. امتیاز گزارش‌دهی، روایی خارجی، روایی داخلی (خطا) و روایی داخلی به ترتیب بین 9-5، 3-1، 4-3 و 5-3 متفاوت بود. نتیجه‌گیری: بسیاری از تحقیقات بیانگر بهبود سیستم تعلیق این نوع از پروتزها نسبت به سیستم‌های رایج بودند. همچنین در خصوص کیفیت راه رفتن، تفاوت محسوسی مشاهده نگردید. در مجموع می‌توان گفت که تحقیقات بیشتری به صورت تست‌های کلینیکی جهت درک بهتر مزایای پروتزهای سیلیکونی در افراد قطع عضو زیر زانو مورد نیاز است.
